# Advantages and Pitfalls in Fluid Biomarkers for Diagnosis of Alzheimer’s Disease

**DOI:** 10.3390/jpm10030063

**Published:** 2020-07-17

**Authors:** Syed Haris Omar, John Preddy

**Affiliations:** 1 Rural Clinical School, Faculty of Medicine, University of New South Wales, Wagga Wagga, NSW 2650, Australia; j.preddy@unsw.edu.au; 2 Murrumbidgee Local Health District, NSW Health, Wagga Wagga, NSW 2650, Australia

**Keywords:** Alzheimer’s disease, cerebrospinal fluid, amyloid beta peptide, total tau, phosphorylated tau, diagnosis

## Abstract

Alzheimer’s disease (AD) is a commonly occurring neurodegenerative disease in the advanced-age population, with a doubling of prevalence for each 5 years of age above 60 years. In the past two decades, there has been a sustained effort to find suitable biomarkers that may not only aide with the diagnosis of AD early in the disease process but also predict the onset of the disease in asymptomatic individuals. Current diagnostic evidence is supportive of some biomarker candidates isolated from cerebrospinal fluid (CSF), including amyloid beta peptide (Aβ), total tau (*t*-tau), and phosphorylated tau (p-tau) as being involved in the pathophysiology of AD. However, there are a few biomarkers that have been shown to be helpful, such as proteomic, inflammatory, oral, ocular and olfactory in the early detection of AD, especially in the individuals with mild cognitive impairment (MCI). To date, biomarkers are collected through invasive techniques, especially CSF from lumbar puncture; however, non-invasive (radio imaging) methods are used in practice to diagnose AD. In order to reduce invasive testing on the patients, present literature has highlighted the potential importance of biomarkers in blood to assist with diagnosing AD.

## 1. Introduction

Alzheimer’s disease (AD) is one of the most common neurodegenerative disease in an ageing population. AD is the most common cause of dementia and is characterized by cognitive impairment and the impedance of daily activities, including communication, decision making and behavioral changes [1]. It has been shown that the frequency of AD doubles for each 5 years of life above the age of sixty years. It is predicted that by 2050, 130 million globally will be symptomatic from AD [2]. In terms of risk factors, advanced age is the most important risk for the sporadic or late onset of AD as well as the presence of APOE e4 alleles. Inherited mutations in chromosome 11 amyloid precursor protein (APP), Presenilin-1 (PSEN_1_), and Presenilin-2 (PSEN_2_) are prevalent in the less common familial form of AD. In addition, women are more prone to AD compared with men, early menopause is also risk factor for AD. Cardiovascular disease and diabetes mellitus type-2 are associated with an increased risk of AD. The exact pathophysiology of AD is still under investigation; however, the deposition of senile plaques, neurofibrillary tangles (NFTs), and astrogliosis are cardinal features [3]. Moreover, studies have shown that pathological involvement of oxidative stress, neuron degeneration induced synaptic alteration, inflammation and microgliosis are important in the pathogenesis of AD [4]. Despite almost 3 decades of research into the exact molecular mechanism causing AD, unfortunately, none of the hypothesis completely answers the question. The still amyloid cascade hypothesis suggests a core pathological role of amyloid beta in AD [5]. The presence of Aβ peptides in cerebral and peripheral tissues mainly consists of amino acids and their sequences ranging from 1 to 43. Aβ_42_ is very prone to aggregate and proceed to form the senile plaques found in hippocampus, neocortex and in the cerebrovasculature region [6]. Another highly aggregated peptide called tau (which undergoes extensive hyperphosphorylation) is responsible for the formation of neurofibrillary tangles inside neurons and ultimately results in extensive brain and nerve damage [7]. Currently, approved drugs only provided symptomatic relief for patients with AD without modifying the disease or slowing disease progression. However, for the treatment of mild cognitive impairment (MCI), there is no FDA-approved drugs available and suggested to consider off-label treatment, such as an acetylcholinesterase (AChE) inhibitor, which has provided a modest impact but is also associated with the risk of side effects. In order to reduce the side effects, research has been undertaken to modify the chemical moiety of drugs with compatible substitutes and also focused on natural products with the potential to act as disease modifying agents [8,9]. Several natural products including curcumin, ginkgolides, resveratrol, oleuropein etc. have been shown to be effective against AD pathology in vitro or in vivo models but have not shown success in randomized trails [10,11,12,13]. Lifestyle modification, including exercise and dietary modification, especially the Mediterranean diet (MedDi) and Mediterranean-Dietary Approaches to Stop Hypertension (DASH) diet Intervention for Neurological Delay (MIND) diet, have been associated with improved cognition among elderly subjects [14].

It has been shown that pathological changes of AD occur long before the appearance of clinical symptoms. Therefore, it is important to establish a diagnosis as early as possible especially for people above the age 60 years. Biomarkers offer essential tools for AD diagnosis, monitoring, early detection, therapeutic intervention, as well as prevention of inaccurate diagnoses. Body fluid biomarkers in cerebrospinal fluid (CSF) and blood have shown potential for AD diagnosis, individual prognosis and patient stratification. Despite the availability of numerous theoretical and clinical diagnostic tools, AD is still poor diagnosed, especially in the early stage of the disease. AD has a prolonged pre-symptomatic prodromal phase; however, the lack of specific biomarker, procedural and methodological inconsistencies, inconsistent cut-off values as well as a lack of assay standardization, have thwarted attempts to establish a diagnosis and treat AD during this early phase.

## 2. Search Methods

Potential studies were identified in electronic database PubMed, Embase, ScienceDirect, Cochrane Library, SpringerLink, Scopus and Google Scholar using combination of following keywords “Alzheimer’s Disease”, “biomarkers” and “Alzheimer’s disease”, “cerebrospinal fluid”, “CSF”, “invasive biomarkers”, “non-invasive biomarkers”, “plasma biomarkers”, “blood biomarkers”, “plasma amyloid”, “plasma tau”, “inflammatory biomarkers”, “imaging biomarkers”, “proteomic biomarkers”, “salivary biomarkers”, “olfactory biomarkers” and “ocular biomarkers”. Selected studies published between 1990 and May 2020 were included to ensure that all randomized trial, pilot studies, and critical reviews or systematic reviews published evidence on potential Alzheimer’s disease biomarkers for three decades were encompassed. The preclinical studies, in vitro studies, published media, as well as duplicate articles were excluded due to being outside the scope of the clinical study aim.

## 3. Biomarkers in Alzheimer’s Disease

**Figure 1:**
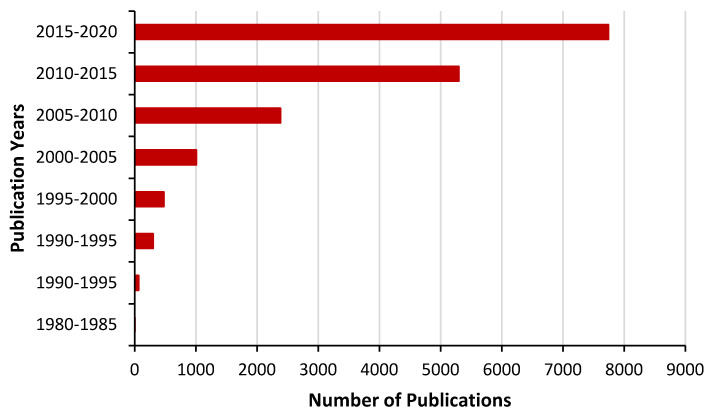
Publications statistics for “Biomarkers in Alzheimer’s disease”, source PubMed.

A biomarker is usually characterized by substances (synthetic molecules, specified cells, proteins, enzymes, hormones or genetic material) or imaging finding, which is used as a metric characteristic to indicate the presence of a specific physiological state and may assist with establishing a diagnosis well before a clinical diagnosis can be made. Furthermore, the use of biomarkers is increasingly for assisting with the prognosis and diagnosis of AD, reflected by a tremendous increase in research from 1980 to current time (Figure 1).

On the basis of AD pathogenesis and clinical condition, a set of diagnostic criteria were established in 1984, which was updated by the National Institute on Aging and Alzheimer’s Association (NIA-AA) [15]. The updated NIA-AA guideline was mainly based upon the pathophysiological advancement in clinical, imaging, and research technologies in AD. Similarly, based upon clinical *probable*, *possible*, or *definite* symptoms, National Institute on Neurological and Communicative Disorder and Stroke and the Alzheimer’s Disease and Related Disorders Association (NINCDS-ADRDA) have also published a diagnostic criteria for AD [16]. The clinical conditions of AD are considered to fall into three stages; however, some studies have expanded this to 1–5 or 1–7 stages. Of all the stages of AD, the prodromal period has the longest duration. This has resulted in a revision of NIA-AA diagnostic criteria, which are mainly based upon the identification of biomarkers, including CSF and imaging as valid diagnostic tools [17]. Based on modern diagnosis criteria, three sets of biomarkers are used as diagnostic tools, including Aβ peptide (A), tau peptide (T) and neurodegeneration (N), which are classified as A/T/N framework (Table 1) of AD diagnosis [17].

## 4. Biomarkers Based upon Alzheimer’s Disease Stages

*Stage 1:* assigned to the individuals who do not have functional impairment but might have cognitive impairment, which can only be detected through neuropsychological sensitive instruments. There is increasing evidence that certain biomarkers can predict the pathological changes at an early preclinical phase, namely the presence of amyloid imaging and a reduced CSF Aβ_42_ concentration. Early diagnosis based on biomarkers may assist with the approval of AD treatment, which could provide clinical benefits and improve outcomes [18]. Further trials are required to evaluate the reliability of clinical measurement and access the potential improvement with the drug-placebo conclusion.

*Stage 2:* Presence of biomarkers that predict pathophysiological changes of AD, a subtle cognitive effect, but no functional deficits in the patients, which can be detected with the use of sensitive instruments; however, they do not fulfil the criteria for dementia. According to the FDA guidance, sensitive neuropsychological testing should be considered alongside biomarker changes to diagnose AD stage 2 [18].

*Stage 3:* Pathophysiological biomarkers are present, and patients have started to have difficulty in doing some daily tasks which are measurable. This stage of the disease corresponds with mild cognitive impairment, whereas the first two stages are preclinical.

*Stage 4, Stage 5 and Stage 6:* Pathophysiological biomarkers are present with the consecutive stages of mild, moderate, and severe AD dementia with worsening cognitive impairment.

### Assessment of Stages in AD

From the FDA classification of stages in Alzheimer’s disease, stage 1 and 2 should be considered critical and monitored seriously. However, from stage 3 onwards, AD patients have similar pathophysiological biomarkers and ongoing cognitive decline. There are two basic questions that stem from the stages of AD. Which biomarkers may predict the presence of stage 1 and stage 2 AD in individuals? Secondly, from the treatment perspective, how can we establish the clinical effect of current FDA-approved drugs for patients with stage-1 AD, which is preclinical (based on the presence of amyloid and reduced CSF Aβ_42_ concentration) without evidence of cognitive decline? There is a need to evaluate predictive biomarkers and establish whether changes in biomarkers is a predictor of treatment success.

## 5. Biomarkers through Invasive Diagnostic Methods

### 5.1. Cerebrospinal Fluid Biomarkers

Cerebrospinal fluid (CSF) is a clear liquid that is present in the subarachnoid space and ventricular system of the brain and spinal cord. The volume of CSF in the body varies between 125 and 150 mL. The composition of CSF can demonstrate minor biochemical change in the brain. Currently, CSF is considered an excellent biologic fluid that may contain potential biomarkers for AD, which may be able to identify without going through autopsy or biopsy. Furthermore, the presence and concentration of biomarkers may change in parallel to AD progression. The three most suggestive biomarkers in AD are Aβ, total tau (t-tau), and phosphorylated tau (p-tau) (Table 2). It has been suggested that CSF biomarkers did not vary with severity with stable levels noted in the follow-up patients with clinical AD [19].

#### 5.1.1. CSF Aβ Biomarker

The amyloid isoforms Aβ_40_ and Aβ_42_ concentration in CSF are considered to be the most dependable biomarkers for the diagnosis of the AD disease. The production of both amyloid isoforms Aβ_40_ and Aβ_42_ was 24% higher for mutation carriers than noncarriers in the autosomal dominant AD patients. However, it was suggested that the fractional turnover rate of Aβ_42_ was noted 65% higher in mutation carriers [22]. Interestingly, it was also reported that there is no change in CSF Aβ_40_, while it is present in a 10-fold higher concentration than Aβ_42_ in the CSF of AD patients. It was suggested that Aβ_42_ be used as a proxy of total Aβ concentration. The amyloidogenic protein is found throughout the human body, and studies showed that Aβ_42_ concentrations in CSF often correlate with Aβ levels in the patient’s brain [23]. It was found that the Aβ_42_ concentration was significantly reduced in CSF, which is a consequence of its presence in fibrils and plaques in the brains of patients with AD [24,25,26]. There are variations in quantification; however, it was found that Aβ_42_ concentration declined by 50% in CSF of patients with AD as a result of its deposition in the brain parenchyma [27].

The underlying mechanisms of the reduction CSF Aβ_42_ is not clear; however, few studies have suggested that it is due to the excessive hydrophobic aggregation of Aβ_42_ sequestration in plaques, a reduction in its diffusion from interstitial fluid to CSF and/or decreased Aβ clearance as a consequence of an impaired blood–brain barrier [28,29]. It was reported that the other isoform of amyloid peptides, Aβ_38_, was also found to have an increased concentration in CSF. Furthermore, the ratio of Aβ_42_/Aβ_38_ closely corresponds with imaging findings in patients with AD and thus results in a robust biomarkers for AD pathogenesis, which is more useful than the concentration of Aβ_42_ alone in CSF [30,31]. In contrast, several studies have reported that the Aβ_40_ concentration was unchanged in CSF from patients with AD and does not correlate with amyloid deposits in the brain [32]. In spite of the discrepancy in the diagnosis of CSF Aβ_40_ levels, several studies supported a decrease CSF Aβ_42_/_40_ ratio in the diagnosis of MCI patients compared to controls [33]. Because of the observed Aβ isoforms ratio and their positive relationship with AD pathogenesis, the NIA-AA has accepted Aβ_42_ concentration as well as a comparative ratio of Aβ_42_/Aβ_40_ as important biomarkers in the diagnostic guideline for AD [17].

#### 5.1.2. Assessment of CSF Aβ_42_ Biomarker

CSF Aβ_42_ biomarkers support a diagnosis of AD in its preclinical stage and are predictors of disease progression in cognitively unimpaired individuals and in those with MCI. One of the main limitations of CSF sampling is its invasive collection technique i.e., through lumbar puncture compared with blood sampling. Post lumbar puncturing, headache as most common adverse effect.

Most of the studies showed a significant decline in Aβ_42_ levels as diagnostic biomarker and agreed that upto 40% reduction was observed in AD patients when compared with those of healthy individuals [34]. In contrast to the reduction of Aβ_42_ concentration in CSF, some of the past studies have shown an unchanged Aβ_42_ concentration [35] and an elevated level [36] in CSF Aβ_42_ concentration compared to AD patients and healthy controls. CSF Aβ_42_ was found to reach the plateau state early in the disease progression and produce a conflicting outcome, which demonstrates a process of preceding aggregation of Aβ mainly detected with amyloid PET analysis. There is a lack of standard protocol and universal agreement because of the varying biomarker concentrations and contradictory outcomes, which required further investigation in the age- and stage-matched individuals considering prodromal stage individuals with different ethnic groups. It has been demonstrated that CSF Aβ_42/40_ ratio may predict abnormal cortical amyloid deposition (visualized with PET) compared with CSF Aβ_42_. However, this diagnosis could result as false positive (low CSF Aβ_42_) or false negative (high CSF Aβ_42_) in fewer patients [25]. Further studies have been reported the presence of oligomers formation prior to the formation of Aβ fibrils in the pathogenesis of AD and suggested oligomers as potential early target in the prodromal stages, which required further confirmation in randomized trial as early diagnostic biomarker.

#### 5.1.3. CSF Tau Biomarker

The Aβ and tau peptides have been suggested to interact mutually and prompt both aggregation and toxicity followed by proposed mechanisms including Aβ encourage tau pathology or tau induces Aβ toxicity, or synergistic toxicity exists between Aβ and tau [37,38]. Basically, tau is a microtubule associated protein have a pivotal action in intracellular transportation. Tau proteins classified as p-tau representing hyperphosphorylation, and t-tau representing several isomers of the tau protein. It was suggested that the hyperphosphorylation occur at threonine-231 (p-tau_231_), threonine-181 (p-tau_181_), and at serine-199 (p-tau_199_). The involvement of p-tau in the assembly of neurofibrillary tangles represented as ‘T’ marker, and their presence in the CSF proving a sign of neuronal death as ‘N’ marker [39,40]. It was reported that CSF p-tau_231_ was involved in the neurofibrillary neocortical pathology [40], and showed a significant increase in concentration correlated with a decline in cognitive performance and conversion to AD [41]. The concentration of t-tau in CSF was found to be highly age dependent and observed <300 pg/mL in 21–50 years, <450 pg/mL in 51–70 years, and <500 ng/L in 71–93 years age group of normal cognitive healthy individuals [42]. Several lines of studies have supported the results of significant rise in CSF tau peptide concentration in AD patients [39,43]. In particular, p-tau and t-tau were found to be increased by 200% 300% concentration in AD compared with nondemented elderly subjects [27,44]. Tau pathology cause an elevated level of CSF t-tau and p-tau and strongly associated with cognitive decline compared to the amyloid pathology.

Furthermore, the degree of neurodegeneration and neuronal/axonal damage in AD patients’ brains marked by the presence of considerable CSF t-tau concentrations and constituted in the A,T,N Framework as a marker of ‘N’ [17,45,46]. A systematic review included 15 studies showed the presence and accuracy of CSF t-tau in seven studies, while six studies have showed the presence and accuracy of the CSF p-tau in mild cognitive impaired patients [47].

The intermediate filaments known as neurofilament light (NfL) were found to be present in the axons cytoplasm and may interfere with cytoplasmic function of axonal homeostasis as well as synaptic transmission. Studies have shown the presence of elevated NfL in AD patients and suggested end results of neuronal and axon damages [48]. More pronouncedly, the increase level of NfL could serve as a risk factor for MCI. However, elevated NfL levels were also recognized as a biomarker in other neurodegenerative diseases having marked axonal degeneration, white matter injury, or both, such as frontotemporal dementia, amyotrophic lateral sclerosis, Creutzfeldt–Jakob disease, multiple sclerosis and traumatic brain injury. A few post-mortem studies have reported significant elevated NfL levels in patients with amyotrophic lateral sclerosis and frontotemporal dementia more than the AD patients and suggested that NfL could be used for differentiation of two types of dementia [49]. Due to non-specificity in disease diagnosis, NfL is less popular as confirmatory biomarkers compared to Aβ and tau in AD.

#### 5.1.4. Assessment of CSF Tau Biomarker

The increase in both CSF t-tau and p-tau concentrations are well settled in AD compared with controls, specifically the intensity of neuronal injury and neurodegeneration are indicated by CSF t-tau in AD. The concentration of CSF t-tau was reported two to three folds higher in patients with AD compared to the normal controls [34]. Still, it is not confirmed about the agreement in distinct tau phosphorylation sites for AD. Most studies have showed the significant rise in tau concentration with aging as well as in patients with AD; however, few studies have reported contradictory information and showed no significant change in the CSF tau level in normal healthy aged individuals [50]. It was postulated that the incidence of tau phosphorylation and the building of neurofibrillary tangles inside neuron is a results of cellular protective mechanism against oxidative stress and suggesting normal physiological pathway rather than a toxic pathway [51]. It is still unknown about the inconsistent results for tau analysis and required to develop a new technique for consistent outcome. It has been suggested that, due to the presence of heterogeneity in trial subjects, the consistency of the result varies and not being reproducible. Thus, there is a requirement to agree on one productive model for the diagnosis of CSF tau biomarkers and run the trial in a large cohort that can be reproducible or verify in a repetitive/confirmatory trial.

### 5.2. Blood Biomarkers in AD

Blood is a most commonly accessible biological sample than other body fluids such as CSF and offer inexpensive clinical diagnosis or screening methods and even convenient for getting reproducible results in clinical trials. In cardiovascular disease and cancer diagnosis and research, biofluid blood has been established as biomarkers; therefore, it may perform as a critical measure in the early diagnosis of AD [52]. Due to the presence of Aβ in the prodromal stage of AD and their ability to pass through the blood–brain barrier, for diagnostic purposes, Aβ received a considerable amount of attention as a potential blood biomarker.

#### 5.2.1. Plasma Aβ Biomarker

In order to evaluate and understand amyloid clearance, studies reported a significant decline in amyloid clearance using the stable isotope labelling kinetic method in late-onset AD patient [53]. Both Aβ_40_ and Aβ_42_ production were found to be increased by 24% in individuals with mutation carriers than noncarriers autosomal dominant form of AD (ADAD), and, furthermore, the Aβ_42_ fractional turnover rate was 65% faster deposition in mutation carriers individuals [22]. Several studies have reported the existence of Aβ in blood plasma and showed the rise of both Aβ_40_ [54] and Aβ_42_ [55] levels. In contrast, studies have also reported a fall in both Aβ_40_ [56,57] and Aβ_42_ [58] concentrations in individuals susceptible to AD. More recently, a comprehensive meta-analysis study showed the inconsistency in plasma for both Aβ_42_ and Aβ_40_ in AD [59]. In order to achieve consistency and accuracy in the biomarker analysis, trials based on immunoprecipitation-mass-spectrometry-based assays for evaluation showed a significant decline in both Aβ_40_ and Aβ_42_ concentration in plasma Aβ_42_/Aβ_40_ ratio (in the line with CSF test) with approximately 90% of diagnostic accuracy [60,61].

#### 5.2.2. Assessment of Plasma Aβ Biomarkers in AD

Blood considered as highly complex fluid connective tissue containing cellular components and several compounds, such as proteinases nature compounds, genetic materials, and metabolites, appear in plasma. The primary barrier for inconsistency in the biomarkers analysis results was suggested due to the presence of low blood Aβ concentration as well as victims of matrix effects. In addition, a lack in assay sensitivity, specificity and methods selectivity are responsible for inconsistency in Aβ finding as biomarkers in blood. In general, biomarkers localized in the brain are not easily available in blood because of the restriction of movement through the blood–brain barrier, poor expression of AD pathology biomarkers in blood and the interference of blood containing endogenous antibodies with the assay reagents that finally resulted in a false rise and fall of measurement. There is a need to develop the analytical sensitive plasma-based assay, which can minimize the event of reaction of biomarkers such as Aβ with the reagent, and careful validation work. A few attempts have been made to analyze Aβ in blood through a new diagnostic technique; however, such attempts were unable to resolve the cerebral expression of Aβ including plasma protein and blood platelets [62] but still represent an important step forward.

#### 5.2.3. Plasma Tau Biomarker

Studies have been reported that the elevated plasma tau levels but with overlapping ranges of results across diagnostic groups of AD patients compared with the normal control [63]. It has been suggested that the plasma tau is a late-stage marker of AD and did not show any change in the plasma of the MCI-stage individual followed by missing the interrelationship between tau levels in plasma and CSF due to the differential regulation of tau in both fluids [63]. Further study showed a positive associations between increased plasma tau level and AD hallmarks [64]. The elevated level of plasma total tau and pTau_181_ were investigated in patients with dementia compared to the cognitively unimpaired individuals [65]. In general, total tau protein concentration was found to be approximately seven times higher than p-tau_181_ in human plasma. Based on immunomagnetic reduction technique, a study evaluated the concentration ratios of p-tau_181_ to t-tau in plasma are 14.4% for healthy controls, 13.6% for patients with MCI due to AD, and 19.5% for very mild AD, respectively, and suggested that p-tau_181_ in plasma can be used to differentiate memory disorder/cognitive decline in early-stage AD patients [66]. Further study based on the evaluation of plasma p-tau_181_ as a biomarker using ultrasensitive immunoassay methods showed a significantly higher plasma p-tau_181_ level in the AD group compared with the control group [67]. A recent study measured plasma-phosphorylated tau concentration and found a significantly higher concentration in the AD group compared with age-matched cognitively normal controls [68]. Tau hyperphosphorylation-induced neuronal damage was also investigated and suggested the presence of neurofilament as biomarker for neurodegeneration in AD [69]. Neurofilament was measured by using an ultrasensitive immunoassay method and showed increased serum neurofilament light (NfL) concentration in familial AD prior to symptomatic disease [70]. In the line of the previous report, a recent study outcome showed an early rise of serum neurofilament light in the presymptomatic phase of familial AD and continue to increase in neurofilament light level, respectively [71]. Increased plasma neurofilament light was found in MCI and AD dementia patients compared with controls and correlated with poor cognition [72].

NfL in plasma well determined, reported their elevated levels in the serum of familial AD patients usually appear a decade ahead to the onset of symptom and well correlated with whole-brain atrophy intensity through MRI and an assessment of cognition [73]. In addition, a high level of NfL was determined in plasma of MCI compared to AD patients and healthy controls, which can be used as a determinant assay to easily distinguish individual between MCI and AD [72].

#### 5.2.4. Assessment of Plasma Tau Biomarkers in AD

In the past two decades, it is still in debate that tau pathology starts early in normal cognitive individuals upon ageing (>60 years) than Aβ pathology. However, research based on tau pathophysiology have showed high consistency in the increase level of tau protein (neurofilament light, p-tau_181_, and total tau as biomarkers) and easily detected in serum. In contrast, Aβ (Aβ_40_, Aβ_42_ and Aβ_42_/Aβ_40_ as biomarkers) protein appearance in blood do not show those consistent results in early stage of AD progression regardless with the determination techniques. It was suggested that ultrasensitive immunoassays granted the accurate quantification of tau in blood. Further research is required to justify and validate tau protein as an early diagnostic biomarker in AD.

## 6. Biomarkers through Non-Invasive Diagnostic Methods

### 6.1. Cognitive Biomarkers

Currently, non-invasive diagnostic criteria for AD based on a group of assessments, including individual clinical history, cognitive and neuropsychological state and clinical rating score resulted from Mini-Mental State Examination (MMSE), Clinical Dementia Rating (CDR) and the Wechsler Memory Scale (WMS) Logical Memory (LM) test [74]. According to the MMSE score (0–30), if an individual has received a score between 20 and 24, then suggested mild dementia, followed by a score between 13 and 20 suggest moderate dementia and have a score less than 12 designated severe dementia [75]. A study evaluated the accuracy of the MMSE for diagnosing dementia subtypes in people aged 65 years who do not examine earlier for dementia and supported the diagnostic use of MMSE as part of the process for deciding whether or not someone has dementia [76]. The LM test consist of LM-I (immediate recall), LM-II (delayed recall), and LM Recognition (delayed recognition), used to investigate and measure verbal episodic memory in individuals [77]. The LM-I directed individual to immediately recall details of two short passages, while LM-II phase based on recall the passages after a 20 to 30-min delay. The LM recognition phase test provided questionnaire-based evaluation on an earlier provided passage in the form of yes or no.

### 6.2. Assessment of Cognitive Biomarkers

Several studies have indicated the psychometric limitations of MMSE analysis, including large ceiling and floor effects, and sensitivity to practice effects, limiting the clinical efficacy of MMSE in MCI and AD dementia investigation. Moreover, the scoring system in MMSE was found to lack accuracy in the investigation of individuals with MCI or mild AD dementia. The LM subtest is not only useful for distinguishing certain types of dementia such as AD dementia but is also known as a tool that can detect subtle memory changes in the individuals with MCI [78].

### 6.3. Imaging Biomarkers

The imaging of the brain in the diagnosis of AD has been used as a second line of diagnostic criteria, including magnetic resonance imaging (MRI), functional MRI (fMRI) and positron emission tomography (PET). In a clinical setting, current guidelines follow the structural imaging, i.e., magnetic resonance imaging (MRI) or computerized tomography (CT), mainly required for the evaluation of patients presenting with a cognitive/dementia syndrome [79]. In order to investigate the visualization of AD-linked cortical atrophy and changes in brain connectivity, MRI is used to provide a structural and functional imaging technique [80]. In addition, fMRI investigation provided the functional connectivity of the brain such as abnormality in the hippocampus [81]. Positron emission tomography (PET) is an advanced imaging technique using compounds labelled with short-lived positron-emitting radionuclides to detect Aβ associated metabolic activity and plaques deposition in AD [82]. Commonly used PET tracers, including Pittsburgh Compound-B (PiB) and Fluoro-2-deoxy-D-glucose (FDG), have a high sensitivity and specificity of diagnosis, particularly in the early stages, and are utilized in imaging biomarkers of amyloid plaque progression in individuals [83].

### 6.4. Assessment of Imaging Biomarkers

In a clinical setting for AD diagnosis, imaging techniques are often used as a second or third line of investigation due to the unavailability of imaging facility at every center, especially in rural areas. Imaging require rigorous measurement, expertise to interpret the findings and burden of high cost, preventing their frequent use in the routine clinical assessment of individual during their first visit. ^18^F-FDG-PET analysis may differentiate dementia from normal aging and is used as an indicator biomarker for neurodegeneration; however, it is unable to track down pathology at an early stage of AD. In addition, the current practicing guidelines and expert opinions suggested that amyloid PET analysis was unable to anticipate the trajectory of AD disease progression for an individual patient [84,85]. Moreover, it is also uncertain how effective the PET analysis is at characterizing differences across the pathophysiological phase of AD.

## 7. Promising Biomarkers in AD

### 7.1. Proteomic or Enzymatic Biomarkers in AD

In order to investigate novel protein and their capacity to predict AD, an early study analyzed 120 plasma proteins and discovered 18 signaling proteins, which showed 90% of accuracy in diagnosis for AD patients and 91% for MCI patients [86]. Further studies have been reported a total of 1590 AD-related proteins, including 296 proteins encoded with 115 up-regulated and 181 down-regulated genes, and supposed to be blood-secretory proteins involved in the pathogenesis of AD [87]. It was suggested that around 35 AD-related proteins are consistent, including four key proteins (APP, apolipoprotein E, PSEN-1, and PSEN-2) involved in AD pathology [87]. Synaptic proteins such as synaptosomal-associated protein 25 (SNAP-25) and synaptotagmin-1 (SYT1) were found to be significantly increased in the CSF of AD dementia and prodromal AD patients; however, SNAP-25 and SYT1 were specified to decline in cortical areas [88,89]. To facilitate early diagnosis in AD, a protein profiling of blood samples in mild AD patients showed a downregulation of apolipoprotein A_1_, α-2-HS-glycoprotein, and afamin, while, apolipoprotein A_4_ and the fibrinogen gamma chain were identified upregulated [90].

The CSF BACE1 enzymatic activity and protein concentration has been elevated in AD patients [91], and represented as an indicator biomarker of MCI [92]. In support of elevated BACE1 activity, a study reported a significant rise in plasma BACE1 activity by 53.2% in subjects with MCI and by 68.9% in patients with AD and suggesting plasma BACE1 activity as a diagnostic biomarker [93]. The study focused on plasma-based biomarkers showed a significantly elevated level of BACE1, and soluble forms of APP were observed in AD patient [94]. Further study showed the presence of increased BACE1 enzymatic activity along with the increase in phospholipase-A_2_ activity in platelets and brains of patient with AD [95]. One of the main interests for determining BACE1 biomarker is the development of specific BACE1 inhibitors, which may help in the reduction of amyloid production. It is unfortunate that the present plasma BACE1 investigation as biomarkers have not demonstrated a consistent result in terms of significant rise in BACE1 in AD patients compared to normal individuals. In search of other proteomic or enzymatic biomarker, the protein kinases such as glycogen synthase kinase 3β (GSK-3β) has been observed in tau protein hyperphosphorylation, and reported significantly elevated in plasma of MCI and AD patients compared with aged-matched controls [96] as well as their elevated level was also observed in white blood cells of AD and MCI patients compared with healthy individuals [97].

### 7.2. Assessment of Proteomic or Enzymatic Biomarkers

Several studies have shown the presence of proteins in plasma or serum, including albumin, lipoproteins, Aβ autoantibodies, fibrinogen, immunoglobulin, apolipoprotein-J, apolipoprotein-E, transthyretin, α-2-macroglobulin, serum amyloid p-component, plasminogen and amylin were found to be strongly interferes with the estimation of specific protein biomarkers. It was found that studies that have been conducted on proteomic or enzymatic biomarkers were only in cohorts with unmatched age patients and a target single protein or enzymatic marker; therefore, such studies were required to be focused on proteomics and enzymatic research in individuals with MCI and AD compared to aged-matched controls. Proteomic or enzymatic biomarkers could be an excellent and potential candidate for blood biomarkers as diagnostic tools, but due to the presence of low concentration and the incidence of protein-protein interactions in plasma result the inconsistency in outcomes.

Therefore, it is difficult to replicate due to the lack of specificity and accuracy involved in the current methods of investigation. Thus, the development of highly sensitive and reproducible novel methods that might have the capability to detect the plasma biomarkers in low concentration with accuracy is required.

### 7.3. Inflammatory Biomarkers in AD

Inflammation is one of the major cellular events considered at the initial pathogenic factor causing neurodegeneration in AD mainly through the activation of microglia. Out of the several investigated inflammatory biomarkers, the diminished C-reactive protein (CRP) level and increased level of triggering receptor expressed on myeloid cells-2 (TREM-2) were observed in the CSF of the patient with AD compared to the normal elderly controls [98]. Moreover, the reduced levels of plasma CRP was also observed in AD patients compared to MCI or normal cognition individuals [99]. The estimation of TREM-2 in CSF and blood is a marker of microglia response. Several studies have investigated the concentration of TREM-2 and reported their increased levels in CSF of AD patient, which was mainly associated with tau pathology compared to the controls [100,101].

Further studies have shown polymorphism in about 23 cytokines, and their 13 types were found to be observed in AD pathogenesis, including interleukins, TNF-α, TGF-β and IFN-γ [102]. Pro-inflammatory cytokines such as TNF-α level were significantly increased followed by decrease in the anti-inflammatory cytokine TGF-β level in CSF, which showed a positive correlation with a higher risk of disease progression from MCI to AD [103]. The concentration of cytokine I-309 level was found to be increased in CSF and suggested as a possible predictor of progression from MCI to AD patients [104]. It has been suggested that the presence of cytokines elevated steadily or reached the highest level upon progression from MCI to AD individuals, including IL-1β, IL-6, TNF-α, IL-18, monocyte chemotactic protein (MCP)-1 and IL-10 can be used as a predicting biomarker for early diagnosis [102].

Microglial activation involved in the stimulation of astrocytic expression of YKL-40 (also known as chitinase-3-like protein-1 (CHI3L1)), which was observed in significantly higher levels in the CSF of AD patients compared to the cognitively normal individuals [105]. Most of the studies have investigated the elevated effect of YKL-40 in CSF; however, less studies have been able to detect YKL-40 in blood, showing a similar finding of increased YKL-40 level in plasma [106].

### 7.4. Assessment of Inflammatory Biomarkers in AD

Despite the elevation of the inflammatory biomarker TREM-2 in AD, few studies have reported conflicting results with no significant change in CSF TREM-2 levels in MCI and AD patients compared to the controls [107]. In addition, to recognize TREM-2 as a potential inflammatory biomarker in AD diagnosis, their physiological function has been questioned whether it has a constructive or a harmful effect in human body. Due to non-specific diagnosis of TREM-2 in other neuroinflammatory diseases, the biomarkers have faced challenges and required further studies to answer these questions on the molecular basis. The other biomarker YKL-40 investigation in AD have shown a limited diagnostic value due to their non-specific findings in neurological disease other than AD. However, it has been suggested that YKL-40 has a potential role in astroglial activation and the assessment of neuroinflammation treatment, which required further investigation with a specified sensitive method and an inflammatory biomarker in AD.

The majority of studies investigated inflammatory cytokines or chemokines as biomarkers insufficiently sensitive in plasma and could not be reproduced. In addition, there was a lack in the conduction of study in a large cohort using imaging or CSF samples of individuals with MCI and AD for the detection of inflammatory biomarkers. Therefore, it is warranted to investigate and confirm inflammatory biomarkers targeting individuals for early intervention in future studies.

### 7.5. Oral, Ocular and Olfactory Fluid Biomarkers in AD

Body fluid other than CSF and blood, including oral, ocular and olfactory have received attention for detecting the biomarkers of AD because of their easily available, noninvasive nature and inexpensive sample collection methods. A decade ago, one pilot study reported a significant rise in saliva Aβ_42_ levels in patients with mild AD compared to the control, while saliva Aβ_40_ level was detected unchanged [108]. Further study reported a two-folds increase in salivary Aβ_42_ concentration in AD patients compared to the controls, which have identical levels of salivary Aβ_42_ regardless of sex or age [109]. Further pilot study using the 1H NMR metabolomics technique for detecting salivary Aβ_42_ found a significant rise in salivary Aβ_42_ concentration in MCI and AD patients compared to controls [110]. In the line of previous results showing the elevated level of salivary Aβ_42_, a study based on enzyme-linked immunosorbent-type assays methods reported an increased Aβ_42_ level in patients with AD compared to controls [111].

In order to attempt early diagnosis and the development of a non-invasive method for AD, studies have reported the presence of significant Aβ plaque concentration deposited in the retina [112]. A quantitative and histological study showed a two-fold increase in retinal Aβ deposition in the form of protofibrils and fibrils among AD patients versus controls [113]. A similar study has detected retinal Aβ plaque deposition two months prior to their presence in the hippocampus and cortices of AD murine models and suggested the appearance of Aβ in the retina is an early event in the pathogenesis of AD [113]. The emerging single visit, label-free, cost-effective eye scan along with the emergence of mobile imaging modalities for the early detection of Aβ plaques in retina through the polarization imaging of retinal Aβ and retinal fluorescence lifetime imaging ophthalmoscopy have been suggested as early diagnostic tools [114].

The olfactory dysfunction has been associated with tau pathology from early to advanced AD and is well recognized. A study based on the Braak stage of AD showed the presence of tau pathology 90% and Aβ 9% in stage 4 followed by 44.6% of tau pathology and Aβ 9% in stage 3 [115]. The olfactory tau pathology in stage 2 represented by 36.4% without Aβ deposition; however, Braak stages 0 and 1 do not show any positive association of tau pathology [115]. Further study showed significant elevation in t-tau protein and p-tau tau protein levels in the patients with AD suffering from loss of smell, compared to the healthy controls [116]. The results of a recent meta-analysis suggested that the identification of olfactory dysfunction was more profound in AD patients compared to MCI patients [117].

### 7.6. Assessment of Oral, Ocular and Olfactory Fluid Biomarkers in AD

The oral, ocular and olfactory biofluid as potential biomarkers in AD diagnosis are still in the preliminary stage of research, requiring further investigation to overcome the methodological heterogeneity and discrepancy in accuracy. Thus, there is a need to develop a reliable quantitative method among consecutive or random samples to determine these biomarkers as an effective tool for diagnosing AD.

## 8. Conclusions

**Figure 2:**
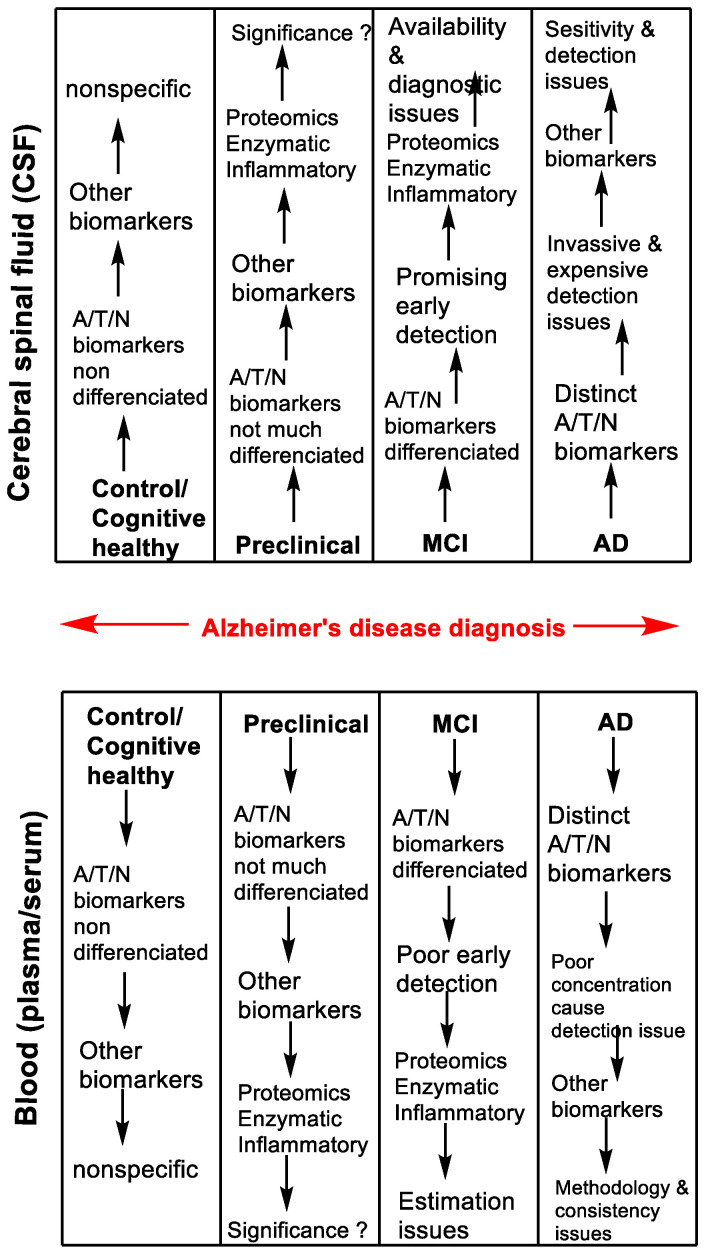
Overview of investigation and development CSF and blood-based biomarkers in Alzheimer’s disease diagnosis.

AD is a multifactorial disease without a confirmatory biomarker; however, based on the NIA-AA guideline, the currently available biomarkers may provide a positive direction to identify individuals who are on risk of AD, and need of routine screening for early diagnosis. The most studied and practiced biomarkers for AD diagnosis are CSF Aβ_42_, CSF Aβ_42_/Aβ_40_, CSF p-tau, amyloid PET, tau PET, structural MRI and fMRI. The accumulated evidences have been established that Aβ_42_ and tau levels were found quite lower (~30 to 100 times) in plasma compared to CSF. Furthermore, because plasma and serum already enriched with immense level of several proteins (~50–70 g/L) which interferes in detection and face challenges in poor outcome of NIA-AA suggested biomarkers in blood compared to CSF. Therefore, there is no doubt that the investigation of biomarkers in blood face inconsistency in findings due to their low appearance (concentration) in blood (plasma or serum) than in the CSF. To date, there are weak evidences supporting the dominance of biomarker over the other (CSF/imaging) for diagnostic tools in AD. Thus, in terms of availability or diagnosis, CSF have less challenges in the A/T/N framework-based biomarkers compared to blood-based biomarkers (Figure 2). Despite of challenges in blood-based biomarkers, the early detection of biomarkers (plasma NfL) could be a promising diagnostic tool in body fluid blood which can be routinely investigate in the individual reaching the age of 60 years. In a nut-shell, blood-based biomarkers should pay more attention in order to support the patient’s comfort, highly sensitive, least invasive and cost-effective inexpensive technologies for biomarker detection are required for detection and analysis in the range of 10^−15^ to 10^−12^ M in individuals before entering MCI stage.

## Figures and Tables

**Table 1:** Biomarkers based upon National Institute on Aging and Alzheimer’s Association (NIA-AA) classification [17].

NIA-AA Classification	Alzheimer’s Disease Biomarkers	Biomarkers Significance in AD
Amyloid (A) aggregates	CSF Aβ_42_, Aβ_42_/Aβ_40_ ratio & Amyloid PET	↓ CSF Aβ_42_ & Aβ_42_/Aβ_40_
Tau (T) aggregates	CSF phosphorylated tau & Tau PET	↑ CSF p-tau
Neurodegeneration (N)	CSF total tau & Anatomic MRI FDG PET	↑ t-tau

NIA-AA: National Institute on Aging and Alzheimer’s Association; ↑: increase; ↓: decrease; CSF: cerebrospinal fluid; Aβ: β-amyloid; PET: positron emission tomography; FDG: fluorodeoxyglucose; MRI: magnetic resonance imaging.

**Table 2:** Established diagnostic biomarkers in the cerebrospinal fluid CSF of Alzheimer’s disease (AD) ^a^ [20] and showed 85% sensitivity cutoff values for AD dementia diagnosis [21].

Biomarkers	Controls (pg/mL)	AD (pg/mL)	% Sensitivity (AD-Control)	% Sensitivity (MCI-Control)
Aβ_42_	794 ± 20	<500 *	73 (≥75 years)	60 (≥75 years)
tau peptide	136 ± 89 (21–50 years)	^b^	74 (≤64 years)	65 (≤64 years)
	243 ± 127 (51–70 years)	>450	53 (65–74 years)	49 (65–74 years)
	341 ± 171 (>71 years)	>600 *	61 (≥75 years)	46 (≥75 years)
p-tau-181	23 ± 2	>60	37 (≥75 years)	30 (≥75 years)

^a^ Data obtained using innogenetics single 96-well ELISA kits. ^b^ This is not relevant for sporadic AD, because it is only for patients >60 years of age. * *p* < 0.001.
